# Conservative Treatment for Childhood and Adolescent Obesity: Real World Follow-Up Profiling and Clinical Evolution in 1300 Patients

**DOI:** 10.3390/nu13113847

**Published:** 2021-10-28

**Authors:** Gabriel Á. Martos-Moreno, Julián Martínez-Villanueva Fernández, Alicia Frías-Herrero, Álvaro Martín-Rivada, Jesús Argente

**Affiliations:** 1 Departments of Pediatrics & Pediatric Endocrinology, Hospital Infantil Universitario Niño Jesús, E-28009 Madrid, Spain; gabrielangelmartos@yahoo.es (G.Á.M.-M.); jmvfernandez@gmail.com (J.M.-V.F.); alicia_fh96@hotmail.com (A.F.-H.); amartinrivada@gmail.com (Á.M.-R.); 2 La Princesa Research Institute, E-28009 Madrid, Spain; 3 Department of Pediatrics, Universidad Autónoma de Madrid, E-28049 Madrid, Spain; 4 Centro de Investigación Biomédica en Red de Fisiopatología de la Obesidad y Nutriciόn (CIBEROBN), Instituto de Salud Carlos III, E-28029 Madrid, Spain; 5 IMDEA Food Institute, CEI UAM & CSIC, E-28049 Madrid, Spain

**Keywords:** childhood obesity, attrition rate, follow-up, success rate, metabolically healthy

## Abstract

**Background:** Limited therapeutic tools and an overwhelming clinical demand are the major limiting factors in pediatric obesity management. The optimal protocol, environment, body mass index (BMI) change targets and duration of obesity-oriented interventions remain to be elucidated. **Aims:** We aimed to characterize the singularities of follow-up, anthropometric and metabolic evolution of a large cohort of pediatric patients with obesity in a specialized university hospital outpatient obesity unit. **Patients and methods:** Follow-up duration (up to seven years), attrition rate and anthropometric and metabolic evolution of 1300 children and adolescents with obesity were studied. An individualized analysis was conducted in patients attaining a high level of weight loss (over 1.5 BMI-SDS (standard deviation score) and/or 10% of initial weight; *n* = 252; 19.4%) as well as in “metabolically healthy” patients (*n* = 505; 38.8%). **Results:** Attrition rate was high during the early stages (11.2% prior to and 32.5% right after their initial metabolic evaluation). Mean follow-up time was 1.59 ± 1.60 years (7% of patients fulfilled 7 years). The highest BMI reduction occurred in the first year (−1.11 ± 0.89 SDS, *p* < 0.001 in 72.5% of patients). At the end of the follow-up, improvements in glucose and lipid metabolism parameters were observed (both *p* < 0.05), that were highest in patients with the greatest weight reduction (all *p* < 0.01), independent of the time spent to achieve weight loss. The pubertal growth spurt negatively correlated with obesity severity (*r* = −0.38; *p* < 0.01) but patients attaining adult height exceeded their predicted adult height (*n* = 308, +1.6 ± 5.4 cm; *p* < 0.001). “Metabolically healthy” patients, but with insulin resistance, had higher blood pressure, glucose, uric acid and triglyceride levels than those without insulin resistance (all *p* < 0.05). Preservation of the “metabolically healthy” status was associated with BMI improvement. **Conclusions:** Behavioral management of children with obesity can be effective and does not impair growth but is highly conditioned by high attrition. The best results regarding BMI reduction and metabolic improvement are achieved in the first year of intervention and can be preserved if follow-up is retained.

## 1. Introduction

Management and follow-up of children and adolescents affected with obesity are crucial clinical challenges worldwide due to both high prevalence and the limited available therapeutic resources. Additionally, obesity management is highly influenced by a number of barriers both, from the patient’s side (stigma, gaps in medical education, misperceptions of the disease or weight status, etc.) and from the healthcare system (insufficient staff, time and facilities to provide timely and patient/family-personalized assistance). These barriers strongly impair the probability of therapeutic success [1].

Despite the recent approval (June 2021) by the European Medicine Agency of liraglutide as the first drug for obesity treatment above 18 years of age, management of obesity in childhood and adolescent largely remains based on combined nutritional and exercise behavioral counseling, ideally involving a multidisciplinary intervention [2,3]. Although primary care assistance is recommended for childhood obesity management, the low rate of successful weight loss in these patients and the increasing prevalence of obesity associated comorbidities has resulted in obesity being one of the most frequent causes for consultation in specialized pediatric endocrinology or obesity clinics in our environment, thus generating increasing delays in patient attention [4]. Nevertheless, there is no consensus regarding the best environment to manage childhood obesity to achieve a high success in weight loss and its maintenance with, for example, primary care assistance allows for higher accessibility and proximity to patients [5] while tertiary care centers normally have more available resources and the possibility to develop multidisciplinary care units [6].

In contrast, there is robust consensus and evidence that the major factor limiting success in childhood obesity management programs is the high withdrawal rate observed throughout follow-up at all levels of assistance [7], reaching up to 92% after 2 years in some reports [8], with sociodemographic and anthropometric features being potential predictors of a higher risk of attrition [9,10]. Among these factors, the perception that obesity is not a disease state [9], initial weight loss wrongly assumed as “curation” [9,10] and, most importantly, lack of sustained weight loss [11], have been postulated as the main risk factors for early termination of intervention programs in children and adolescents with obesity. Consequently, the combination of some of these factors (early weight loss + high drop-out rate + lack of sustained weight loss), can result in selection bias in the analysis of the results of these programs in the long term.

An additional concern of parents upon intervention for childhood obesity is the potential effect on growth and pubertal development of their child. These patients usually exhibit some degree of advancement in skeletal maturation and overgrowth in relation to their target height [12,13] and this is directly correlated with the severity of their obesity and inversely proportional to the magnitude of their pubertal growth spurt [13] but does not impair the attainment of their predicted adult height [13,14,15]. Consequently, although severe caloric restriction is not usually included in childhood and adolescent obesity interventions [2,3] and evidence indicates that conventional strategies for weight control in this age range do not impair growth or puberty [15], this can be a factor underlying the reluctance of some parents to intervene.

Some points of discussion in childhood obesity management protocols include the degree of weight loss needed to achieve clinically significant improvements, the role (if any) of the time to attain weight loss and the amount of time this BMI reduction should be sustained for in order to preserve the beneficial effect of the intervention. A decrease in BMI Z-score of 0.25 or more has been suggested to be sufficient to improve cardiometabolic risk factors [16], whereas a duration over 3 years of intervention, beginning at the earliest age possible, has been associated with more successful outcomes [17]. However, there is limited knowledge regarding what should be considered an excellent degree of weight reduction under conservative treatment and what is an acceptable evolution after attaining this degree of weight loss [18].

A subgroup of patients with obesity that has raised special interest are those classified in some studies as “metabolically healthy” [19]. This concept of “metabolically healthy” in children with obesity was inherited from the concept first postulated in adults where a patient with excess fat mass, but normal blood pressure, lipid profile and glycemia was proposed to be metabolically healthy [20]. However, the evidence that insulin resistance is the first step of carbohydrate metabolism impairment in childhood obesity, often preceding the rise of blood glucose levels [12,21,22] and the data supporting the role of uric acid as a potential marker of metabolic impairment in children [23] have lead to the question of whether these parameters should also be considered before assuming that a child with obesity is “metabolically healthy” [20,21,22].

Based on the above observations, we hypothesized that the time required to obtain a significant reduction in BMI affects both the attrition rate and the degree of improvement in metabolic impairment in children with obesity. Thus, the aims of this study were: (1) To analyze the duration of follow-up, the drop-out rate and its causes and the behavioral changes implemented, along with the patient’s BMI Z-score, growth and metabolic evolution up to a maximum of 7 years in the regular outpatient care in an obesity clinic in a tertiary hospital, with a particular emphasis on BMI changes and attrition rate. (2) To study the features of patients achieving a large reduction in BMI (“excellent responders”), characterizing their weight loss and metabolic changes, the evolution of their BMI in the 5 years following weight reduction and exploring how the time required to attain weight loss affects the metabolic changes observed. (3). To compare the features of patients with obesity, with or without metabolic comorbidities during their first evaluation, as well as the evolution of their metabolic status according to BMI changes during follow-up, while evaluating the role of insulin resistance in the definition of a “metabolically healthy status”.

## 2. Patients and Methods

### 2.1. Study Cohort

One thousand and three hundred children and adolescents [mainly Caucasians (75.8%) and Latinos (19.0%)] with standardized BMI above +2 SDS for national and international references [24,25] were enrolled during a period of 6 years after potential underlying pathological or syndromic causes of obesity were ruled out. Patients above 12 years of age and their parents or guardians gave informed consent as required by the local ethics committee, which had previously approved the study in accordance with the “Ethical Principles for Medical Research Involving Human Subjects” adopted in the Declaration of Helsinki by the World Medical Association.

At the first visit of all patients (baseline, B) weight, height, BMI, pubertal status and systolic and diastolic blood pressure were recorded and standardized when indicated [26]. A left wrist/hand X-ray was used to estimate bone age according to the Greulich & Pyle method [27] and a 12-h fasting serum sample (serum stored at −80 °C until assayed) was used to determine glucose, insulin, HbA1c (hemoglobin A1c), the lipid profile and uric acid levels by standardized assays and to calculate the HOMA homeostatic model assessment) index as previously reported (cohort characterization displayed in Table 1) [12].

Patients were always seen in the outpatient clinic in our department by the same physician (GAM-M). Visits were scheduled one month after baseline, every three months during the first year and every six months thereafter up to 7 years for the maximum follow-up. Treatment consisted of lifestyle reorganization (dietary and exercise related behavioral counseling) mainly focused on three key elements: avoidance of snacking and sweetened drink consumption, establishing a slow pace of food intake in meals and onset of scheduled daily physical activity. A daily recommendation for food group distribution was provided on a weekly basis in addition to the categorization of usual foods as recommended, non-recommended and allowed with limited frequencies/amounts. Self-monitoring of the fulfillment of the key elements of lifestyle reorganization was encouraged and specific documents for fulfillment registration provided.

Time of follow-up, drop-out rate and its causes, along with the patients’ BMI Z-score, growth and pubertal evolution throughout follow-up were studied and their last available anthropometric and metabolic evaluation prior to the end of their follow-up were compared with those at baseline. To study the metabolic evolution, the last available metabolic analysis was used and only those patients that had an analysis at least 12 months after their baseline evaluation were included (available in 451 patients).

### 2.2. Excellent Responder Group

Two hundred and fifty-two patients (19.4% of the entire cohort) achieved a reduction in their BMI over 1.5 SDS and/or in their weight over 10% from baseline. Anthropometric and biochemical features for each patient after weight loss were compared with those at baseline, studying the eventual role of the time to attain weight loss on the observed changes. Additionally, the evolution of BMI in the 5 years after weight loss was analyzed.

### 2.3. Metabolically Healthy Group

According to their baseline features, patients were classified as: (1) “Metabolically healthy” (**MH**; *n* = 505; 38.8%) if they showed normal blood pressure, lipid profile (low density and high density (LDL-c, HDL-c) lipoproteins and triglycerides), and serum glucose and uric acid levels at diagnosis or (2) “Not metabolically healthy” (**No-MH,** *n* = 795, 61.2%) if one or more of these parameters were impaired. Additionally, patients were classified according to their fasting insulin levels as insulin resistant (**IR**, fasting insulin ≥ 15 µU/mL; *n* = 481; 37%) or **No-IR** (fasting insulin < 15 µU/mL *n* = 819, 63%).

Standardized BMI, blood pressure, fasting lipid profile, glucose, insulin, uric acid levels and HOMA index at baseline were compared between **MH** and **No-MH** patients, also accounting for the presence or absence of **IR**. In addition, the evolution of these features was studied by longitudinally comparing status at baseline and the end of follow-up, considering the eventual role of the background of **IR** at diagnosis.

### 2.4. Statistical Analysis

Data are shown as mean ± SD. For normally distributed parametric variables, comparison between two independent groups was performed using Student’s *t* test, whereas *t*-test for related measurements was used for comparing patient features between different timepoints. For those parametric variables with non-normal distributions, the Mann–Whitney U test and the Wilcoxon test were used. To compare non-parametric variables, Chi square tests (independent groups) and McNemar tests (paired samples) were used. The relationships between quantitative normal variables were studied by linear correlation analysis (Pearson’s r), whereas Spearman’s rho was used for non-normally distributed variables. A value of *p* < 0.05 was chosen as the level of significance. The software used was Statistical Package for Social Sciences (SPSS v. 15.0. MapInfo Corporation, Troy, NY, USA).

## 3. Results

### 3.1. Follow-Up Characterization

#### 3.1.1. Time of Follow-Up and Drop-Out

**Figure 1:**
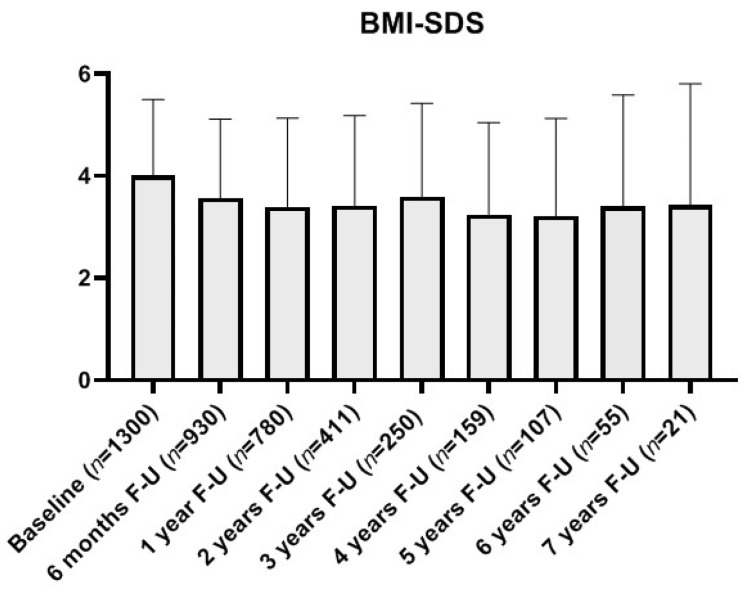
Evolution of patient retainment and their standardized BMI (expressed as SDS) throughout follow-up to a maximum of 7 years from their first evaluation. *Abbreviations:* BMI-SDS: Standardized body mass index (Z-score); F-U: Follow-up; n: number of patients retaining follow-up.

Mean duration of follow-up in this cohort was 1.59 ± 1.60 years, with 59.9% patients stopping it unilaterally and 33.5% requesting the end of follow-up in their last visit (mainly due to the achievement of BMI or metabolic improvement). A progressive decrease in the study population was observed throughout follow-up with only 21 patients (7% of initial cohort) completing 7 years of follow-up (Figure 1). The drop-out rate before 6 months of follow-up was higher in Latinos (52% vs. 24% in Caucasians; *p* < 0.001), males (36% vs. 23% in females; *p* < 0.05) and prepubertal (32% vs. 26% in pubertal; *p* < 0.05). After 1 year, the interethnic difference in attrition rate persisted (47% in Latinos vs. 18% in Caucasians; *p* < 0.001). Among the patients dropping out, 84.1% had repeatedly not fulfilled the therapeutic recommendations in the visits prior to withdrawal and 5.4% argued familial/social difficulties. A total of 8% of the patients re-consulted after follow-up had stop (most after regaining lost weight).

It is of note that, among the patients abandoning follow-up, 11.2% did it after the initial clinical examination (not performing the complementary tests indicated) and 32.5% after their second visit once they received the results from the complementary examinations (total 43.7% patients abandoned prior to their third visit).

#### 3.1.2. BMI Evolution and Lifestyle Changes

**Figure 2:**
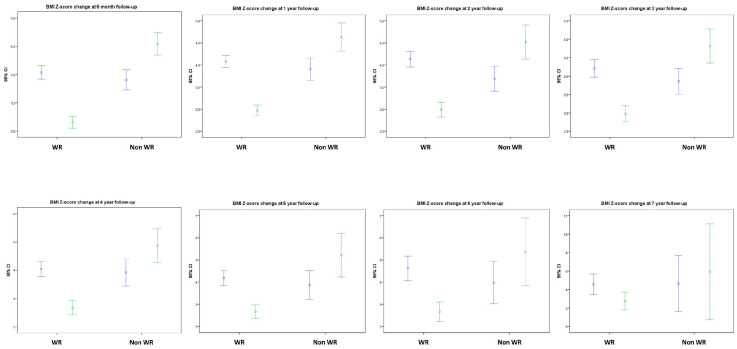
Evolution of BMI SDS at each time-point shown as a paired comparison with patients’ BMI Z-score at baseline (taking into account exclusively those continuing follow-up at each timepoint) differentiating between those with weight reduction (WR) or increasing (Non WR) their BMI. *Abbreviations:* 95% CI: Confidence Interval 95%; Non WR: Not showing weight (or BMI) reduction; WR: Showing weight (and BMI) reduction. The percentages of patients showing WR among those retaining follow-up were, respectively: 66.8% at 6 months, 72.5% at 1 year, 71.6% at 2 years, 68.0% at 3 years, 73.1% at 4 years, 78.1% at 5 years, 72.2% at 6 years and 80.0% at 7 years (See Figure 1 for the number of patients in each time point).

In the analysis of BMI evolution at 6 months, 66.8% of patients of follow-up had reduced their BMI (−0.89 ± 0.73 SDS), whereas 32.5% had increased it (+0.56 ± 0.53 SDS, both *p* < 0.001), reducing the mean cohort BMI in 0.41 ± 0.95 SDS compared to **baseline** (*p* < 0.001). At 12 months, mean cohort BMI was −0.63 ± 1.16 SDS below the initial BMI (*p* < 0.001), with 72.5% of the patients at follow-up showing BMI reduction from **baseline** (−1.11 ± 0.89 SDS, *p* < 0.001). After a significant decrease in mean cohort BMI in the first year of follow-up, a partial regain was observed at the second year, with later stabilization (Figure 1 and Figure 2).

The mean BMI at the end of follow-up (*n* = 980, +3.59 ± 1.87 SDS) was lower than BMI at baseline (−0.37 ± 1.25 SDS; *p* < 0.001) with 62.65% patients having achieved some degree of BMI reduction (−1.04 ± 0.88 SDS; *p* < 0.001) but with 36.43% of patients increasing their BMI despite assistance (+0.79 ± 0.93 SDS; *p* < 0.001). At the end of follow-up, 83.1% of patients still maintained their BMI above +2 SDS (obesity), with 10.3% shifting to the overweight category (BMI centile 90 to 97) and 6.6% normalizing their BMI (below centile 90).

The percentage of patients acknowledging snacking (eating in-between meals) at their baseline visit was 81.9% and this was reduced significantly (*p* < 0.001) at their second (55.9%) and last visit (57.2%). A similar pattern was observed for compulsive eating behavior (50.6% at the second visit and 47.3% at the last visit vs. 74.0% at baseline, *p* < 0.001). Additionally, the number of patients who did not perform any scheduled physical activity prior to their enrollment was 74.7%, with this improving significantly (*p* < 0.001) at their second (53.1%) and last visits (49.8%) (Table 2).

#### 3.1.3. Metabolic Evolution

Metabolic data at the end of follow-up were available for 451 patients and showed a significant decrease in HOMA index (−0.35 ± 2.07; *p* < 0.001) with a positive correlation between the parallel decrease in HOMA and BMI-SDS (r = +0.15; *p* < 0.01). This was also observed for triglyceride (−4.99 ± 53.54 mg/dL; *p* < 0.01/r = +0.12; *p* < 0.01) and LDL cholesterol levels (−4.89 ± 17.50 mg/dL; *p* < 0.001/r = +0.19; *p* < 0.001) whereas HDL cholesterol levels increased at the end of follow-up (+2.81 ± 8.72 mg/dL; *p* < 0.001/r = −0.20 with a change in BMI; *p* < 0.001). Despite no significant differences in mean uric acid levels between baseline and the end of follow-up, a direct correlation also existed between the change in uric acid levels and BMI SDS (r = +0.12; *p* < 0.05) as well as a decrease in the percentage of patients showing hyperuricemia (12.6% at the end of follow-up vs. 17.8% at baseline; *p* < 0.05).

#### 3.1.4. Growth and Puberty throughout Follow-Up

In 81 patients (45 females and 36 males), follow-up encompassed their entire pubertal development (from its onset, or Tanner stage II, to its completion and attainment of adult height). Mean duration of puberty in this group was 3.23 ± 1.16 years, with no differences according to sex or race. In girls, the time from puberty onset (Tanner stage II) to menarche was 1.65 ± 0.91 years, again with no ethnicity-based differences. A negative correlation between the duration of puberty and baseline BMI-SDS was observed (r = −0.25; *p* < 0.05).

The pubertal growth spurt in this group was 16.29 ± 5.80 cm, with no differences between sexes and with a negative correlation between the degree of the growth spurt and the severity of obesity at baseline estimated by BMI-SDS (r = −0.38; *p* < 0.01).

In addition to these 81 patients, 227 additional patients achieved their final height during their follow-up (total 308 patients with available adult height). Their mean adult height was above their predicted target height (mean parental height +/− 6.5 for boys and girls, respectively) by +0.29 ± 0.94 SDS (+ 1.6 ± 5.4 cm; *p* < 0.001). In this group, adult height prediction using the Bailey Pinneau method using their bone age at their first visit was shown to overestimate the attained final height by +0.22 ± 0.78 SDS (+ 1.5 ± 4.56 cm, *p* < 0.001). This overestimation was greater in males and prepubertal patients.

### 3.2. The Excellent Responder Cohort

The characteristics of the 252 patients who achieved a reduction in their BMI of over 1.5 SDS and/or of their weight of over 10% from baseline are shown in Table 3. There was a higher proportion of prepubertal children among good responders (χ^2^: 10.57; *p* < 0.01), whereas the relative percentages of the two main ethnicities was similar to those in the cohort at baseline.

The mean weight loss was 3.85 ± 5.92 kg, resulting in a mean BMI decrease of 1.59 ± 0.77 SDS. However, the mean body weight decrease needed to achieve the threshold set was higher in pubertal compared to prepubertal patients (−6.01 vs. −2.01 kg, respectively; *p* < 0.001). After weight loss patients experienced a significant increase in their HDL levels and a significant decrease in glucose, insulin, triglyceride, and LDL cholesterol, as well as in HOMA index (all *p* < 0.01, Table 4).

The mean time from baseline to weight loss achievement was 0.94 ± 0.86 years, with 35.3% of patients attaining this goal at 6 months and 74.2% before 12 months of follow-up. No differences in time to weight loss were observed according to sex or pubertal status.

The longer the time needed to attain BMI reduction, the lower the amount of raw weight loss achieved (r = −0.65; *p* < 0.001). However, no correlation between the magnitude of changes in metabolic parameters and the time spent to achieve weight loss was observed.

**Figure 3:**
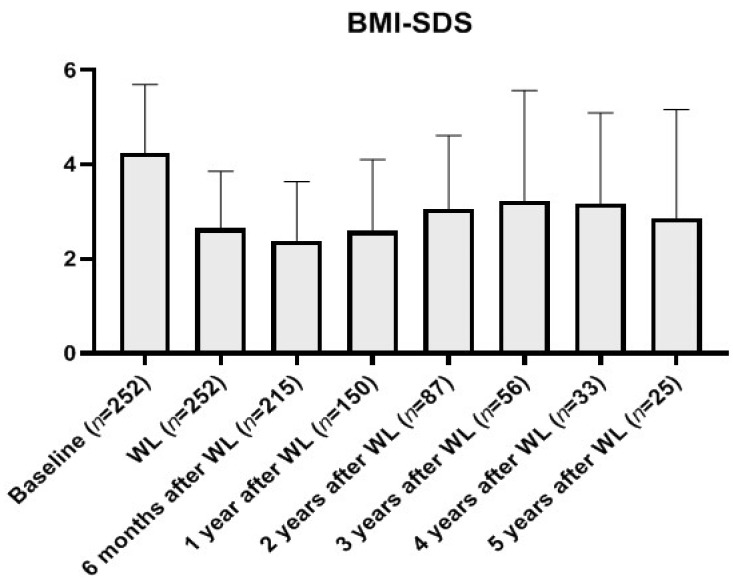
Evolution of patient retainment and their standardized BMI (expressed as SDS) in the five years following weight loss in the excellent responder group. *Abbreviations:* BMI-SDS: Standardized body mass index (Z-score); WL: Weight loss.

**Figure 4:**
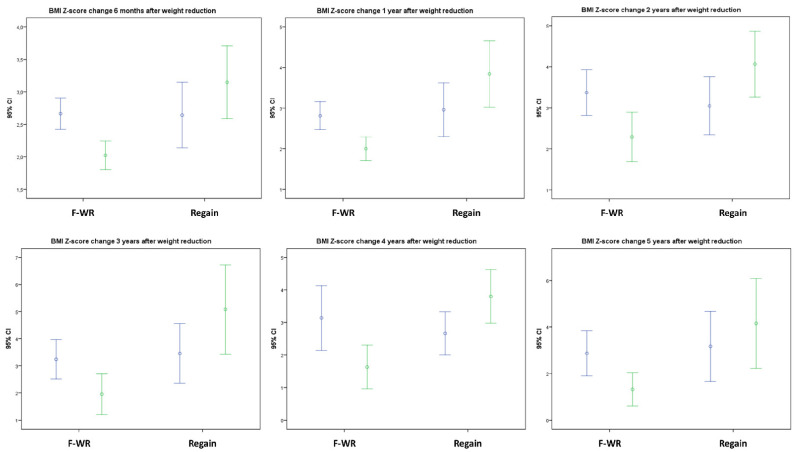
Evolution of BMI Z-score at every time-point throughout the 5 years following intense weight loss in the “excellent responder” group. Shown as the mean of a paired comparison with each patient’s BMI Z-score at each timepoint compared to baseline (taking into account exclusively those retained at each follow-up) differentiating between those further reducing their BMI (F-WR) or regaining it (Regain) after the initial intense weight loss. *Abbreviations:* 95% CI: 95% Confidence interval; F WR: Further weight (and BMI) reduction; Regain: Showing weight (and BMI) increase after initial intense loss. The percentages of patients showing F-WR after initial weight loss among those retaining follow-up were 71.1% at 6 months, 63.9% at 1 year, 53.7% at 2 years, 61.3% at 3 years, 38.9% at 4 years, and 69.2% at 5 years.

Prospective follow-up showed that the BMI reduction was preserved and even enhanced after 6 months upon weight loss attainment but increased from the first to the third year after weight loss and later remained stable in those patients achieving 5 years of follow-up (Figure 3 and Figure 4).

### 3.3. “Metabolically Healthy” Cohort

No differences in age, sex or pubertal distribution between the MH and No-MH groups were observed. However, the MH group showed a slightly lower mean standardized BMI (+3.78 ± 1.30) than No-MH (+4.15 ± 1.59 SDS; *p* < 0.001).

**Figure 5:**
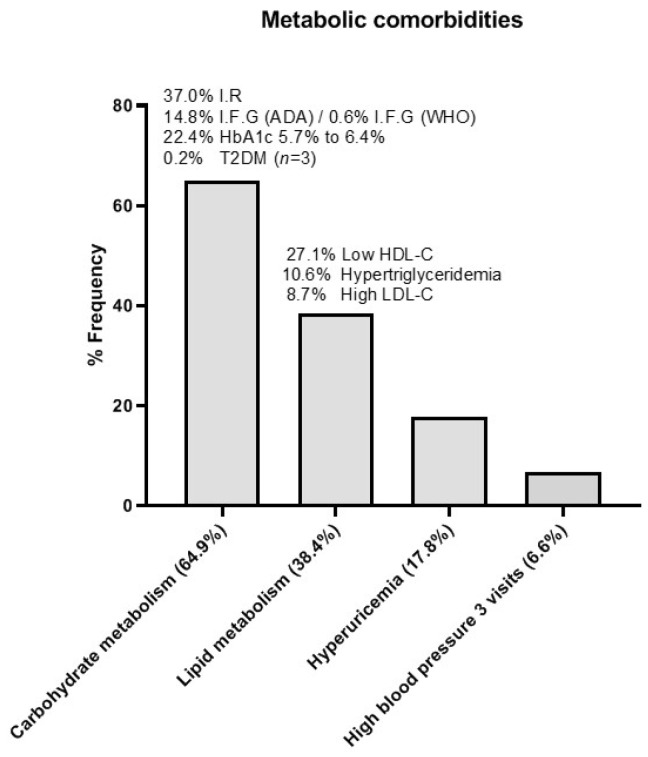
Prevalence of metabolic comorbidities in the entire study cohort at baseline based on fasting determinations. *Abbreviations:* ADA: American Diabetes Association (criteria for IFG: ≥100 mg/dL); HDL-c: High density lipoprotein cholesterol; IFG: Impaired fasting glucose; IR: Insulin resistance (fasting insulin ≥ 15 µU/mL); LDL-c: Low density lipoprotein cholesterol; T2DM: Type 2 diabetes mellitus (ADA criteria: ≥126 mg/dL, confirmed); WHO: World Health Organization (criteria for IFG: ≥110 mg/dL).

The degree of metabolic impairment categories observed in the entire cohort based on a fasting serum sample determinations and using the criteria previously described (12) are shown in Figure 5.

The prevalence of MH patients was lower in Latinos (30.4%) than in Caucasians (41.0%; χ^2^ 9.358; *p* < 0.01) and was also lower in IR (28.1%) compared to No-IR patients (48.0%; χ^2^ 46.003; *p*< 0.01). Consequently, the prevalence of IR (37.0% in the whole cohort) was higher in the No-MH group (44.8%) than in the MH patients (25.5%). Among MH patients, those with IR (*n* = 129) were older, more severely obese, and had higher systolic and diastolic blood pressure and glucose, uric acid and triglyceride levels and lower HDL-c levels than the MH patients without IR (Table 5).

Metabolic data at the end of follow-up were available for 152 of the 505 MH patients at the onset of the study (30%). Among these, 73.7% remained MH whereas 26.3% had developed at least one metabolic comorbidity (independently of the presence or absence of IR at diagnosis). Among the No-MH patients (metabolic data at the end of follow-up were available in 29.8% [237/795]) 8.4% became MH by the end of follow-up, whereas 91.6% still showed at least one metabolic comorbidity.

A significant decrease in BMI from baseline to the end of the study was observed both in those patients who remained MH (+3.50 ± 1.02 to +2.76 ± 1.36 SDS, *p* < 0.001) as well as in those who resolved their initial comorbidities (+3.58 ± 1.57 to +2.69 ± 2.28 SDS, *p* < 0.05), but not in those developing comorbidities throughout follow-up or remaining No-MH.

## 4. Discussion

In this study we observed that a high attrition rate was the most relevant and limiting factor in our outpatient specialized assistance pediatric obesity clinic, with a very high drop-out rate in the early stages of intervention as well as a low mean duration of follow-up. We have seen how the fulfillment of behavioral counselling and the attainment of BMI reduction occurs most frequently in the first year of intervention, with a significant percentage of patients being very successful, and with metabolic improvement being attained independently from the time required to achieve weight loss and that can be sustained if follow-up is retained. We have also seen that controlled intervention does not affect growth or pubertal development, nor does it impair the attainment of the adult target height, although the timing and pace of growth are influenced by obesity and its severity. Finally, we saw that insulin resistance is related to metabolic status in patients with obesity, even before the onset of other metabolic alterations, and should be considered when defining whether a person is metabolically healthy, especially when considering that in childhood obesity the metabolic statis is a dynamic condition related to the evolution of the patient’s BMI over time.

This study, similar to most preceding reports, highlights the evidence and relevance of the high attrition rate in intervention programs for childhood and adolescent obesity [8,9,10,11,28], with the number of patients in follow-up declining over time. Here, we found that 43.7% dropped out before their third visit and only 7% extended their visits up to 7 years. Around 60% of patients unilaterally decided to stop the follow-up. As might be expected, 84.1% of them had not fulfilled the therapeutic recommendations in their visits prior to withdrawal, whereas only 5.4% stated that they had difficulties to attend the programmed visits.

Several factors have been analyzed to predict and avoid a patient’s dropout, including misperception of disease status, ethnicity, socio-economic or cultural determinants or unavailability to attend the visits [7,9,10,11,29]. Among these possibilities, misperception of the parents and children of their weight status or the conception that obesity is not a real disease is particularly important in our [30] and most western environments [31,32]. This underestimation of the pathogenicity of childhood obesity could, at least in part, explain why 11.2% of the patients in our cohort did not even perform the complementary test requested and withdrew after their first interview and clinical evaluation, even though they had been referred for specialized care by their primary care physician. This limited parental and child concern could also be involved in the high rate of patients not fulfilling the recommendations while in follow-up prior to attrition. Additionally, a large percentage of the population assumes that it is the onset of metabolic comorbidities, particularly type 2 diabetes or dyslipidemia, but not weight excess itself that confers the pathogenic potential to obesity [9]. This could influence the additional 32.5% of patients who dropped out at their second visit after getting the results from their metabolic evaluation.

This degree of acceptance of obesity is further enhanced if a positive background of familial obesity exists [31], with more severe obesity and higher prevalence of comorbidities observed in the offspring of parents with obesity at the time of consultation [33], with this being closely related to ethnic, socio-economic and cultural factors [31]. Although the parental academic level background distribution in our cohort was similar to that of the general population in our country [34], no family economic data were registered. These factors could potentially underly the significantly higher attrition rate observed in Latino patients compared to Caucasians at early stages of intervention.

Interestingly, up to one third (33.5%) of the parents asked to discontinue the visits, most after the children had improved their BMI or metabolic comorbidities. This early withdrawal from obesity intervention programs is thought to be a result of the misperception that the obesity was cured [35], which can result in weight regain and later re-consultation as observed in 8% of our patients, most of them after having regained previously lost weight.

Even assuming the high drop-out rate and the positive selection bias derived from a higher attrition rate in those patients not fulfilling therapeutical recommendations, mean follow-up duration in our cohort was similar to that reported in other long-term follow-up series [36], with a retention rate of 54.5% after 1 year; with 72% of the patients showing some BMI reduction (mean over −1 SDS) and with almost 20% being excellent responders (75% of these also in the first year). Subsequently, 31.6% and 15.4% of the cohort extended their follow-up over 2 and 3 years, respectively, resulting in a reduction in mean BMI at the end of follow- up of over 1 SDS in 62% of patients. The degree of fulfillment of the main behavioral recommendations, snacking avoidance, control of compulsive eating and scheduled physical activity, followed a parallel pace to patients’ BMI evolution, significantly improving as early as the second visit and remaining stable to the end of follow-up. This reinforces the relevance of the changes attained in the early stages of the intervention to its final outcomes.

Our data show a higher retention rate and success in weight loss in this cohort compared to previous series [8,36], even though the proximity to the patient’s home and the possibility of a greater frequency of contact, reported to positively influence behavioral outcome [29,37], is limited at the tertiary care level. These results could be influenced by the relevance that the parents place on being sent to specialized care by the primary care physician, the reduced mean age (10.46 years), the severity of the patient’s obesity (mean BMI above +4 SDS) and the high prevalence of metabolic comorbidities in our cohort, all of which could enhance the parental perception of disease in their offspring. Consequently, this could underly the higher rate of excellent responders among prepubertal patients that can be more influenced by their parents’ concerns compared to pubertal patients. Additionally, the development of the intervention in a socialized national healthcare system, with no economic factor biasing visit schedule, and the designation of the same physician for the successive visits of every patient could, among other factors, increase their loyalty and the retention rate once follow-up is settled [38].

The combined analysis of these data suggests that the first year of intervention is crucial to the final outcome and efforts should be focused on the attainment of the maximum BMI change in the first year of follow-up, as this increases the chances of sustaining the achieved weight loss, at least in the following 5 years, as seen in the excellent responder group. However, early weight loss can result in a “double edged sword”; that is, it can be the first step for sustained BMI improvement, but also can determine a misconception of definite success resulting in follow-up withdrawal and weight regain due to the return to unhealthy behaviors, which should be prevented [35]. Thus, rapid weight loss may not be the best option for every patient and an individualized strategy regarding the amount and pace of weight loss should be agreed upon in each singular case. This is supported by two observations: (1) among the excellent responders no correlation was observed between time to attain weight reduction and the magnitude of metabolic changes; (2) the excellent responders that required a longer time to achieve weight loss, thus reducing the influence of weight loss on growth, or those patients achieving a more modest weight reduction, independently of the time spent to attain it, also showed significant metabolic improvement.

Consequently, efforts must be made for the continuation of follow-up to consolidate the decrease in BMI, as its duration is an independent predictor of success [39]. Indeed, a duration of at least three years of follow-up has been proposed [17] and it is consistent with the maximum follow-up time observed for the majority of patients in our cohort. However, at the end of the intervention, only 6.6% of the patients normalized their BMI and 10.3% shifted to overweight, with 82.1% of them remaining above the threshold of obesity (+2 BMI-SDS), which emphasizes the difficulties with reverting to this chronic condition and suggests that the coordinated assistance between specialized care (possessing resources and specialized units) and primary care (having proximity and accessibility) could enhance and prolong the benefits of obesity-oriented interventions [40].

### 4.1. Influence of Intervention and BMI Changes on Metabolic Comorbidities

The effect of BMI reduction on the improvement of metabolic status observed in the excellent responders might be expected. However, the mean decrease in HOMA index and lipid metabolism parameters observed in the 451 patients out of the total 1300 patient cohort reevaluated metabolically prior to the end of follow up, with a mean cohort decrease in BMI of −0.37 DSD, independently of the duration of follow-up, had a linear relationship with the magnitude of BMI decrease, which reinforces the idea that the change in BMI is the primary factor in metabolic health in children and adolescents with obesity. Furthermore, BMI reduction was observed in patients that became metabolically healthy during follow-up or that were already metabolically healthy at the onset of the study and/or at the last clinical visit; however, BMI was not reduced in patients that had persistent metabolic alterations or developed these alterations during the study.

Regardless of the criteria used for defining metabolically healthy in children with obesity [19,20,21,22], the results of this study reinforce the role of insulin resistance as the initial step for metabolic derangement in childhood obesity, even before any analytical abnormality in glucose, uric acid or lipid metabolism is detected [12,19], with metabolically healthy patients with IR showing significant differences in these metabolic parameters compared to those that are metabolically healthy without IR, even though this is not considered in the proposed consensus definition for this entity during childhood [20]. However, the consideration of insulin resistance, next to other elements such as inflammatory markers, adipokine levels or measurements of ectopic fat deposition, has been proposed in more recent revisions of this term [21,22] indicating that more precise analyses of body fat content and distribution (using DXA scan or abdominal MRI) should be considered to better describe the “metabolically healthy” phenotype in obesity. More importantly, our data indicate that during childhood, being classified as metabolically healthily obese is dynamic and mainly dependent on the evolution of the patient’s BMI throughout childhood and adolescence. Additionally, the lower prevalence of metabolically healthy individuals in the Latino patients in our cohort, previously already shown to present higher insulin resistance, triglyceride levels and prevalence of liver steatosis when using our populational standards [12,41], should lead us to consider whether homogeneous standards are valid for every patient, or whether ethnic specific standards for these parameters should be advised.

### 4.2. Influence of BMI on Puberty and Growth

We have confirmed previous observations pointing to accelerated skeletal maturation in children with obesity, resulting in a standardized height over their target height and directly correlated with the severity of obesity [12,13]. Interestingly, the follow up of a significant number of our patients over an extended period of time, some of them encompassing their entire pubertal development and growth spurt, confirms the lack of impairment of obesity-oriented intervention on growth and the attainment of adult height, which was equal to or slightly above that expected according to parental heights [13,14]. Additionally, we observed that the mean pubertal growth spurt in obesity is lower and inversely correlated with the severity of obesity. At the same time the presence of obesity seems to abolish the differences between sexes in the degree of the growth spurt observed in physiological conditions. This could be related to the acceleration in skeletal maturation and the relative childhood overgrowth observed in these patients [12,13]. In contrast, the mean pubertal duration (3.23 years) and time from Tanner stage II to menarche in girls (1.65 years) were similar to that of the general population, despite the inverse correlation observed between obesity severity and duration of puberty. It is clear that several factors such as sex, severity of obesity or the degree of advancement in skeletal maturation determine the features of growth during childhood, and particularly during puberty. This should be taken into account when evaluating height, bone age and growth pace in children and adolescents with obesity as previously reported [12,23].

### 4.3. Study Limitations

The major limitation of this study derives from the fact that patient follow-up was performed during the regular outpatient clinic activity and not in an ideally controlled research design that would result in increased homogeneity in study time-points and data collection for all patients, particularly regarding the final follow-up time-point. Consequently, the high attrition rate observed and the modifications and delays in patient appointments, frequent in daily clinical practice, has resulted in a degree of heterogeneity in data collection. Additionally, the analysis of several obesity-related topics based on the regular activity in the clinical setting with a large number of patients throughout a long timeframe does not allow for the performance of specific examinations (i.e., body composition study) in all patients that would allow for a more thorough analysis of each aspect of auxological or metabolic affectation. However, at the same time this is one of the valuable singularities of this study, which represents the “real world” characteristics and difficulties of daily clinical practice and follow-up in childhood obesity in our environment. The simultaneity of the analysis of BMI evolution, attrition rate, metabolic changes, growth or puberty, though not exhaustive for each topic, will surely be useful for professionals involved in the care of these patients.

## 5. Conclusions

We can conclude that: (1) Behavioral management in an outpatient specialized clinic for childhood and adolescent obesity can be effective in a significant number of patients and it does not impair lineal growth (privative of this age range and absent in adult patients) that contributes to the effective achievement of BMI reduction. (2) The outcome of the intervention is conditioned by a high attrition rate whose predictive factors should be considered when individually designing the intervention schedule for each patient. (3) BMI reduction is the main determinant of metabolic improvement in children and adolescents with obesity, with the latter being achieved even after a sustained modest BMI reduction and not related to the time spent to achieve weight loss. (4) The best results in BMI reduction are attained during the first year of intervention, particularly in a subset of patients, and can be maintained if follow-up is retained. However, weight can be regained if intervention is stopped prematurely. Consequently, a sufficient follow-up duration must be ensured for these patients.

In summary, our observations support the concept that in a chronic disease such as obesity, long-term, if not life-long, treatment is often required and that the intervention to improve weight and metabolic status must be started at early ages in an attempt to achieve a healthy adulthood.

## Figures and Tables

**Table 1:** Clinical features of the entire cohort and in the two main ethnicities.

	Total Cohort (*n* = 1300)		Caucasians (*n* = 986/75.8%)		Latinos (*n* = 247/19.0%)	
	Prepubertal	Pubertal	Prepubertal	Pubertal	Prepubertal	Pubertal
*n*	693 (53.3%)	607 (46.7%)	525 (53.2%)	461 (46.8%)	130 (52.6%)	117 (47.4%)
Sex	**F** 263 (38.0%) **M** 430 (62.0%)	**F** 351 (57.8%) **M** 256 (42.2%)	**F** 192 (36.6%) **M** 333 (63.4%)	**F** 271 (58.8%) **M** 190 (41.2%)	**F** 56 (43.1%) **M** 74 (56.9%)	**F** 66 (56.4%) **M** 51 (43.6%)
Age (years)	8.26 ± 2.54	12.96 ± 1.97	8.47 ± 2.38	13.10 ± 1.94	7.68 ± 2.82	12.53 ± 2.05
BMI-SDS	4.21 ± 1.52	3.77 ± 1.43	4.14 ± 1.42	3.71 ± 1.36	4.38 ± 1.84	3.81 ± 1.39
Glucose (mg/dl)	91.65 ± 7.00	93.44 ± 6.70	91.34 ± 7.08	93.27 ± 6.70	92.83 ± 6.58	94.17 ± 6.85
HbA1c (%)	5.45 ± 0.34	5.47 ± 0.30	5.45 ± 0.34	5.45 ± 0.30	5.43 ± 0.34	5.53 ± 0.28
Insulin (µU/mL)	11.78 ± 6.94	17.19 ± 10.74	11.47 ± 6.37	16.51 ± 10.44	13.01 ± 8.46	18.98 ± 11.39
HOMA	2.70 ± 1.65	4.01 ± 2.66	2.63 ± 1.56	3.85 ± 2.63	2.99 ± 1.86	4.43 ± 2.67
LDL-c (mg/dL)	99.72 ± 26.31	93.36 ± 24.27	99.87 ± 26.03	93.56 ± 24.42	101.97 ± 27.58	93.16 ± 24.30
HDL-c (mg/dL)	47.33 ± 11.10	43.93 ± 9.84	47.83 ± 10.85	44.50 ± 9.99	45.34 ± 11.56	40.94 ± 8.51
Triglycerides (mg/dL)	74.64 ± 44.34	85.63 ± 53.60	72.75 ± 43.51	82.69 ± 53.78	82.61 ± 45.90	97.2 ± 54.40
Uric acid (mg/dL)	4.47 ± 0.92	5.28 ± 1.11	4.51 ± 0.89	5.30 ± 1.05	4.30 ± 0.92	5.11 ± 1.12

*Abbreviations:* BMI-SDS: Standardized body mass index (Z-score); F: Female; HDL-c: High density lipoprotein cholesterol; HOMA: Homeostatic model assessment; LDL-c: Low density lipoprotein cholesterol; M: Male; n: Number of patients.

**Table 2:** Key behavioral items at baseline and after intervention.

	Baseline	Second Visit (*p* vs. Baseline)	Last Visit (*p* vs. Baseline)
Snacking	81.9%	55.9% (*p* < 0.001)	57.2% (*p* < 0.001)
Eating compulsivity	74.0%	50.6% (*p* < 0.001)	47.3% (*p* < 0.001)
Lack of scheduled physical activity	74.7%	53.1% (*p* < 0.001)	49.8% (*p* < 0.001)

**Table 3:** Clinical features of the excellent responder group at baseline.

Age (Years)	10.41 ± 3.19
BMI-SDS	4.24 ± 1.46
Ethnicity:	
- Caucasian	78.6
- Latino	18.1
- Others	4.3
Sex (%)	
Female	38.5
Male	61.5
Pubertal status (%)	
Prepubertal	57.10 (70.1% males/29.9% females)
Pubertal	42.90 (50.0% males/50.0% females)

*Abbreviations:* BMI-SDS: Standardized body mass index (Z-score).

**Table 4:** Changes in BMI and metabolic parameters after weight loss compared to baseline.

	Baseline (*n* = 252)	After Weight Reduction (*n* = 252)	
BMI (SDS)	3.99 ± 1.43	2.69 ± 1.21	*p* < 0.001
Glucose (mg/dL)	94.39 ± 7.10	92.20 ± 6.78	*p* < 0.01
Insulin (mcU/mL)	15.25 ± 8.35	10.73 ± 5.28	*p* < 0.001
HOMA index	3.58 ± 2.07	2.47 ± 1.27	*p* < 0.001
Total cholesterol (mg/dL)	151.93 ± 29.99	146.35 ± 30.20	*p* < 0.01
LDL-c (mg/dL)	93.52 ± 27.92	87.76 ± 24.81	*p* < 0.001
HDL-c (mg/dL)	43.66 ± 10.06	46.13 ± 11.70	*p* < 0.001
Triglyceride (mg/dL)	74.03 ± 48.19	63.10 ± 37.58	*p* < 0.001
Uric acid (mg/dL)	5.05 ± 1.20	4.98 ± 1.14	N.S. (*p* = 0.07)
Ferritin (ng/mL)	39.00 ± 20.98	40.63 ± 20.76	N.S.
Total proteins (g/dL)	7.25 ± 0.42	7.24 ± 0.46	N.S.
Albumin (g/dL)	4.09 ± 0.27	4.17 ± 0.28	*p* < 0.01
25 [Vitamin D] (ng/mL)	22.54 ± 5.82	24.45 ± 6.90	N.S.

*Abbreviations:* BMI-SDS: Standardized body mass index (Z-score); F: Female; HDL-c: High density lipoprotein cholesterol; HOMA: Homeostatic model assessment; LDL-c: Low density lipoprotein cholesterol; n: number of patients; N.S.: Not significant.

**Table 5:** Clinical features of metabolically healthy (MH) patients and comparison according to the presence or absence of fasting hyperinsulinemia (Insulin ≥ 15 μU/mL vs. Insulin < 15 μU/mL).

All MH (*n* = 505)	Insulin < 15 μU/mL (*n* = 376)	Insulin ≥ 15 μU/mL (*n* = 129)	
Age (years)	10.14 ± 3.20	11.62 ± 2.58	*p* < 0.001
BMI-SDS	3.66 ± 1.25	4.12 ± 378	*p* < 0.001
SBP (mmHg)	113.46 ± 11.68	118.26 ± 12.57	*p* < 0.001
DBP (mmHg)	58.83 ± 6.89	61.68 ± 6.71	*p* < 0.001
Fasting glucose (mg/dL)	90.35 ± 5.32	91.68 ± 4.73	*p* < 0.05
Uric acid (mg/dL)	4.41 ± 0.79	4.63 ± 0.85	*p* < 0.01
HDL-cholesterol (mg/dL)	51.01 ± 9.15	48.41 ± 7.32	*p* < 0.01
Triglycerides (mg/dL)	55.54 ± 22.10	68.47 ± 24.27	*p* < 0.001

*Abbreviations:* BMI: body mass index; DBP: diastolic blood pressure; SBP: diastolic blood pressure; SDS: Standard deviation score. Data are shown as mean ± SD.

## Data Availability

Data supporting reported results are preserved in investigators’ records and available.
